# Detection of haplosporidian protistan parasites supports an increase to their known diversity, geographic range and bivalve host specificity

**DOI:** 10.1017/S0031182019001628

**Published:** 2020-04

**Authors:** S. A. Lynch, S. Lepée-Rivero, R. Kelly, E. Quinn, A. Coghlan, B. Bookelaar, E. Morgan, J. A. Finarelli, J. Carlsson, S. C. Culloty

**Affiliations:** 1 School of Biological, Earth and Environmental Sciences, University College Cork, Cork, Ireland; 2 Aquaculture and Fisheries Development Centre, Environmental Research Institute, University College Cork, Cork, Ireland; 3 Area52 Research Group, School of Biology and Environmental Science/Earth Institute, University College Dublin, Dublin, Ireland

**Keywords:** Bivalves, cockles, haplosporidia, *Minchinia*, mussels, parasites, protists

## Abstract

Haplosporidian protist parasites are a major concern for aquatic animal health, as they have been responsible for some of the most significant marine epizootics on record. Despite their impact on food security, aquaculture and ecosystem health, characterizing haplosporidian diversity, distributions and host range remains challenging. In this study, water filtering bivalve species, cockles *Cerastoderma edule*, mussels *Mytilus* spp. and Pacific oysters *Crassostrea gigas*, were screened using molecular genetic assays using deoxyribonucleic acid (DNA) markers for the Haplosporidia small subunit ribosomal deoxyribonucleic acid region. Two Haplosporidia species, both belonging to the *Minchinia* clade, were detected in *C. edule* and in the blue mussel *Mytilus edulis* in a new geographic range for the first time. No haplosporidians were detected in the *C. gigas,* Mediterranean mussel *Mytilus galloprovincialis* or *Mytilus* hybrids. These findings indicate that host selection and partitioning are occurring amongst cohabiting bivalve species. The detection of these Haplosporidia spp. raises questions as to whether they were always present, were introduced unintentionally *via* aquaculture and or shipping or were naturally introduced *via* water currents. These findings support an increase in the known diversity of a significant parasite group and highlight that parasite species may be present in marine environments but remain undetected, even in well-studied host species.

## Introduction

The phylum Haplosporidia consists of 36 recognized species in four genera, *Urosporidium*, *Minchinia*, *Haplosporidium* and *Bonamia*. Certain haplosporidian species have been credited with causing some of the most serious epizootic marine disease breakouts on record, in particular in shellfish species (Carnegie *et al*., 2016). In recent years, over ten newly detected haplosporidian species have been added to the phylum, including species in the *Bonamia* and *Minchinia* lineages (Arzul and Carnegie, 2015). In a previous study, environmental samples (water and sediment) from South Africa, Panama and the UK were molecularly screened, and revealed previously undescribed phylogenetic lineages within the Haplosporidia (Hartikainen *et al*., 2014). Besides their low detection prevalence, a major reason for the lack of detection of novel haplosporidian taxa is thought to be the use of (and increasing reliance on) broadly targeted molecular probes that are unsuitable for the highly divergent genes that characterize parasite groups (Hartikainen *et al*., 2014). Despite these obstacles, novel haplosporidians continue to be discovered in host/carrier species and habitats including the recently detected *Haplosporidium pinnae* in the fan mussel *Pinna nobilis* in the western Mediterranean Sea, thus highlighting the possibility that a significant diversity of haplosporidians have yet to be discovered (Lynch *et al*., 2013, 2014; Arzul and Carnegie, 2015; Pagenkopp Lohan *et al*., 2016; Ramilo *et al*., 2017; Catanese *et al*., 2018).

Recent discoveries have highlighted that the geographic range of Phylum Haplosporidia is much greater than originally appreciated. *Bonamia ostreae*, which has caused significant mortalities in the European flat oyster *Ostrea edulis*, was thought to exclusively occur in the Northern hemisphere in both western and eastern North America and Europe but is now known to extend to the southern hemisphere in New Zealand where it has parasitized the native oyster *Ostrea chilensis* (Lane *et al*., 2016). *Bonamia exitiosa* was originally described in *O. chilensis* in New Zealand (Dinamani *et al*., 1987; Hine, 1996) but is now known to have an extensive geographic range in the southern and northern hemisphere and can infect several oyster species (Hill-Spanik *et al*., 2015). *Haplosporidium nelsoni*, the causative agent of MSX disease in the eastern oyster *Crassostrea virginica* in North America, was detected for the first time in Irish and Spanish Pacific oysters *Crassostrea gigas* and in *O. edulis* in Ireland (Lynch *et al*., 2013). In addition, *Minchinia mercenariae*, reported to cause infections in the hard clam *Mercenaria mercenaria* from the Atlantic coast of the United States (Ford *et al*., 2009), was detected in the common cockle *Cerastoderma edule* in the Netherlands (Engelsma *et al*., 2014) and the UK (Longshaw and Malham, 2013) where it was implicated in host population crashes, and an *M. mercenariae*-like parasite was recently confirmed in *C. edule* in Galicia, Spain (Ramilo *et al*., 2017).

Parasites in the phyla Haplosporidia have been reported infecting a number of bivalve hosts from across Europe. Species known to infect *C. edule* are *Haplosporidium edule, Minchinia tapetis* and *M. mercenariae* (Longshaw and Malham, 2013; Ramilo *et al*., 2017) and in mussels *Minchinia* sp. in *Mytilus galloprovincialis* along the Mediterranean coast of France (Comps and Tige, 1997), *Haplosporidium* sp. in *M. edulis* in Maine (Figueras and Jardon, 1991), USA, a haplosporidian-like parasite in *M. edulis* in Atlantic Canada (Stephenson and McGladdery, 2002) and *Minchinia mytili* in *Mytilus edulis* (Ward *et al*., 2019). Lynch *et al*. (2014) assessed the health status of *Mytilus* spp. around the coasts of Ireland and Wales, and detected a previously undescribed haplosporidian (Haplosporidia sp. SAL-2014) belonging to the *Minchinia* clade in a single *M. edulis* from Wales (Lynch *et al*., 2014). The sequence of this haplosporidian was most similar to *Minchina chitonis* detected in the chiton *Lepidochitona cinereus* and an undescribed haplosporidian species parasitizing the Florida marsh clam *Cyrenoida floridana* (Reece *et al*., 2004). *H. nelsoni* has been associated with *C. gigas* populations in California (Friedman, 1996; Burreson *et al*., 2000), Korea (Kern, 1976), France (Renault *et al*., 2000), Japan (Friedman, 1996; Kamaishi and Yoshinaga, 2002) and Ireland (Lynch *et al*., 2013). In addition to *H. nelsoni*, *Haplosporidium costale*, a species associated with seaside organism (SSO) disease in *C. virginica*, was recently detected in *C. gigas* in China for the first time (Wang *et al*., 2010).

The objectives of this study were: determine (1) if Haplosporidia spp. were present in cockles, mussels and oysters at particular sites in Ireland, (2) did coinfection occur and (3) what abiotic and biotic factors associated with site influence and anthropogenic activities, i.e. aquaculture and shipping may influence their presence.

## Materials and methods

### Study sites, bivalve spp. sampled and site description

**Fig. 1.**
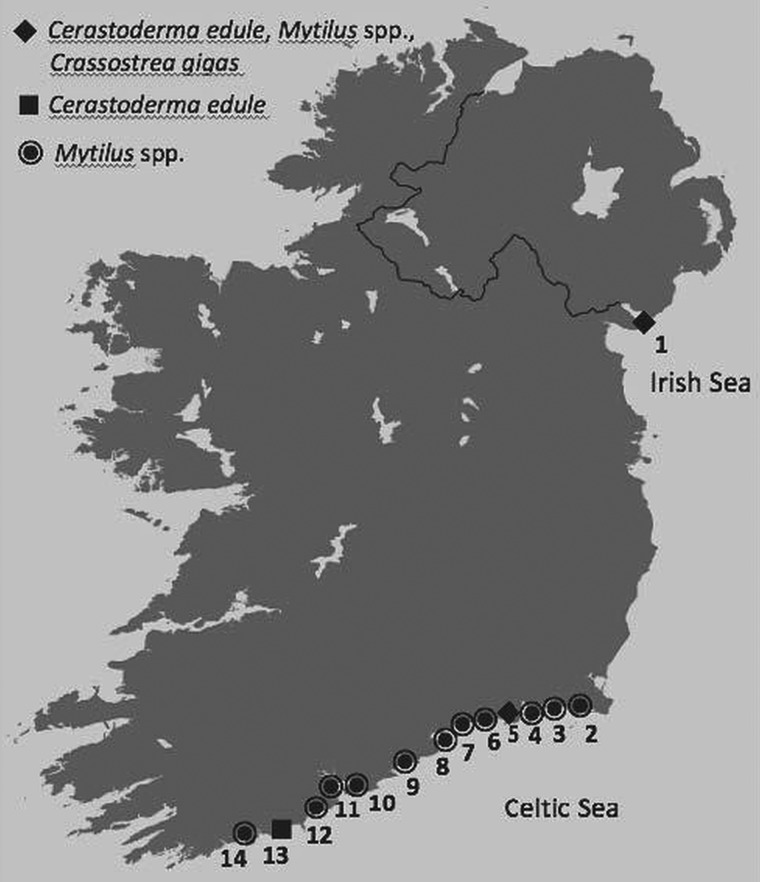
Map of Ireland showing the sample sites and bivalve species sampled and screened for Haplosporidia spp. at each location during this study.

A range of Irish coastal sites was sampled with different environmental (abiotic and biotic) factors and anthropogenic influences (aquaculture and shipping) from nature reserve sites to key economic areas influenced by frequent and heavy anthropogenic effects (Table 1, Fig. 1). Multiple samples of cockles (*n* = 1,604), mussels (*n* = 516) and oysters (*n* = 420) were collected from five of the fourteen Irish sites over several months and years resulting in a higher overall number of individuals being screened at those sites. A minimum sample size of thirty individuals was collected on a single occasion from five other sites.

**Table 1.** Description of bivalve species, sample sites, months, years and anthropogenic activities at each site, and histology and molecular analyses for Haplosporidia spp

Site # (Fig. 1)	Site	Anthropogenic activity	Month	Year	*n*	PCR+	Histology+	Sequencing
			*Wild C. edule*					
13	Flaxfort	Rural/agriculture	March–June	2010 to 2011	900	1.7% (1/60)	0.11% (1/900)	1/Isolate221 (A#KY522823.1)
5	Dungarvan^a^	Aquaculture & agriculture	April–August	2015	250	4% (10/250)	50% (10/20)	1/Isolate226 (A#KY522823.1)
1	Carlingford^a^	Aquaculture & shipping	April–August	2015	454	10.8% (49/454)	100% (20/20)	1/Isolate228 (A#KY522823.1)
	*Total*				*1604*	7.9% (60/764)	3.3% (31/940)	
			*Wild Mytilus* spp.					
1	Carlingford^a^	Aquaculture & shipping	April–August	2015	120	0/120	0/120	
2	Annestown	Rural/agriculture	September	2017	30	0/30	0/30	
3	Stradbally	Rural/agriculture	June	2017	30	0/30	0/30	
4	Clonea	Rural/agriculture	September	2017	60	1.7% (1/60)	1.7% (1/60)	1/Isolate376 (A#KC8828762)
5	Dungarvan^a^	Aquaculture & agriculture	September	2015 & 2017	276	0/276	0/276	
6	Helvic Head^a^	Aquaculture & agriculture	June	2017	30	0/30	0/30	
7	Ballyquinn^a^	Rural/agriculture	June	2017	30	0/30	0/30	
8	Whiting Bay	Rural/agriculture	July	2017	30	0/30	0/30	
9	Ballymacoda^a^	Aquaculture & agriculture	June & November	2017	60	0/60	0/30	
10	Roches point	Shipping		2017	60	0/60	0/60	
11	Ringaskiddy	Urban & shipping	July & October	2017	60	3.3% (2/60)	3.3% (2/60)	
12	Garrettstown	Rural/agriculture	May	2017	70	0/70	0/70	
13	Flaxfort	Rural/agriculture	June	2017	60	0/60	0/60	
14	Lough Hyne^a^	MNR/rural/agriculture	May	2017	60	0/60	0/60	
	*Total*				*976*	2.5% (3/120)	2.5% (3/120)	
			*Cultured C. gigas*					
5	Dungarvan^a^	Aquaculture & agriculture	April–August	2015	180	0/180	0/60	
1	Carlingford^a^	Aquaculture & shipping	April–August	2015	240	0/240	0/60	
	*Total*				*420*			

+ Positive detection.

A# denotes Genbank Accession Number exact match to.

a Special Areas of Conservation (SAC) and Special Protected Areas (SPA) under the birds and habitats EU Directive.

#### Cockle, mussel and pacific oyster samples

Wild *C. edule* was sampled at a nonculture site (March 2010–June 2011) and at two *C. gigas* culture sites (April to August 2015) (Table 1). Cultured *C. gigas* were sampled from both culture sites in 2015. Wild *Mytilus* spp. were sampled from both culture sites in 2015, from a third culture site in September 2017 and from 11 nonculture sites in May to November 2017.

#### Histology

A cross section of tissue (mantle, gill, connective, digestive and gonad) was taken from each cockle, mussel and oyster, and was fixed in Davidson's solution for 24–48 h and subsequently stored in 70% ethanol. The fixed tissue was then dehydrated fixed in paraffin cut at 5 *µ*m and stained using haematoxylin and eosin (H&E) and mounted in dibutyl phthalate xylene. Slides were examined using a Nikon light microscope at 4×, 10×, 20×, 40× and 100× magnification.

#### Molecular genetic diagnostic techniques

Genomic deoxyribonucleic acid (DNA) from *C. edule*, *Mytilus* spp. and *C. gigas* gill tissue (~5 mm^2^ from fresh and fresh frozen (−20 °C)) was extracted from each individual using the Chelex-100 extraction method (Walsh *et al*., 1991).

Several polymerase chain reactions (PCRs) using generic and specific primers and thermocycling conditions for Haplosporidia spp. were utilized in the molecular genetic screening. Additionally, a PCR to detect the nuclear DNA markers Me15/Me16 (Inoue *et al*., 1995) was carried out on mussels that amplified a PCR product to determine if they were *M. edulis*, *M. galloprovincialis* or hybrids of both parent species.

Two generic PCRs using similar mastermix and thermocycling conditions with primer pair HAP-F1′/HAP-R3′ (Renault *et al*., 2000; Lynch *et al*., 2014) and ssu980/HAP-R1′ (Molloy *et al*., 2012; Lynch *et al*., 2014) to amplify the Small subunit ribosomal deoxyribonucleic acid (SSU rDNA) region of Haplosporidia spp. were carried out on *C. edule*, *Mytilus* spp. and *C. gigas* genomic DNA. A third PCR using specific *H. nelsoni* MSX-A′/MSX-B′ primers to amplify the small subunit ribosomal ribonucleic acid (SSU rRNA gene) and similar mastermix and thermocycling conditions (Renault *et al*., 2000) was carried out on DNA from *C. gigas*. Negative controls containing double distilled water (ddH_2_O) were used in each PCR to control for contamination and infected Haplosporidia (*B. ostreae*, *H. nelsoni*, Haplosporidia sp. SAL-2014) genomic DNA was used as a positive control. Initially, in the screening of cockles from 2010/2011 no *M. mercenariae*-like positive material was available as it had not been detected before, however amplification in that single PCR occurred with the cockle deemed positive in the histology and that cockle's DNA was subsequently used as the positive control in the screening of the 2015 samples.

Electrophoresis of amplified products was carried out in a 2% agarose gel and was run with an electrical current of 110 V for 45 min. The expected product size for the HAP-F1′/HAP-R3′ PCR was 350 bp (Renault *et al*., 2000), for the ssu980/HAP-R1′ was 430 bp (Molloy *et al*., 2012) and for the MSX-A′/MSX-B′ PCR was 573 bp (Renault *et al*., 2000).

Pooled PCR products using replicates (×3) from individual cockles [Flaxfort (*n* = 1), Dungarvan (*n* = 1) and Carlingford (*n* = 1)] using the HAP-F1′/HAP-R3′ primers and mussels (Clonea, *n* = 1) using the ssu980/HAP-R1′ primers were used to increase the amplicon concentration for Sanger sequencing, as recommended by EurofinsMWG. Both forward and reverse DNA sequences that were optimally generated by EurofinsMWG Sanger sequencing laboratory were matched against the National Center for Biotechnology Information (NCBI) nucleotide database with Basic Local Alignment Search Tool (BLASTn), which finds regions of local similarity between sequences to identify and confirm the DNA being detected in the PCRs. Percentage query coverage in BLAST refers to how much of the query sequence is aligned with results from the database sequence or, in other terms, the size of the sequence fragments that are comparable, while % identity measures the extent to which the nucleotide sequences relate to one another.

#### Phylogenetic analysis

18S SSU rDNA sequence data for 28 operational taxonomic units from GENBANK (Table 2) were downloaded, to which, the two 18S sequences were added. These data were aligned using Clustal Omega (Sievers *et al*., 2011) at the European Bioinformatics Institute portal (https://www.ebi.ac.uk/Tools/msa/clustalo/). The final alignment was 2221 bp in length. The best-fit evolutionary model for the aligned sequence data was assessed in the jModelTest (v2.1.10; Darriba *et al*., 2012), using the small sample corrected Akaike information criterion (Hurvich and Tsai, 1989). This returned the generalized time reversible (GTR) model, with a four-category gamma rate distribution and invariant sites (GTR + G + I) as the best-fit model.

**Table 2.** Operational taxonomic units from GENBANK used in the phylogenetic analysis

Genbank accession number	Taxon
AF174374	*Massisteria marina*
AF101052	*Cercomonas longicauda*
X77692	*Euglypha rotunda* strain CCAP 1520/1
HGU42447	*Heteromita globosa*
CAU42449	*Cercomonas* sp.
CAU42450	*Cercomonas* sp.
AY449715	Haplosporidian parasite of *Pandalus platyceros*
AY449716	Haplosporidian parasite of *Pandalus platyceros*
AF492442	Haplosporidian parasite of *Haliotis iris*
AY449714	Urosporidium parasite of *Stictodora lari*
UCU47852	*Urosporidium crescens*
HLU47851	*Haplosporidium louisiana*
HNU19538	*Haplosporidium nelsoni*
AF387122	*Haplosporidium costale*
AY452724	*Haplosporidium pickfordi*
AY449713	*Haplosporidium lusitanicum*
AF262995	*Bonamia ostreae*
AF192759	*Bonamia ostreae*
AF337563	*Bonamia* sp.
AF508801	*Bonamia roughleyi*
AY449710	*Minchinia tapetis*
FJ518816	*Minchinia*_*mercenaria*
KY522821	*Minchinia* sp. Clone 1
KY522823	*Minchinia* sp. Clone 3
AY449711	*Minchinia chitonis*
MTU20319	*Minchinia teredinis*
AY449712	Haplosporidian parasite of *C. floridana*
EF165631	*Minchinia occulta*
	Isolates 221, 226 & 228 – this study, isolate 376 – this study

The phylogeny was reconstructed in Mr Bayes (v3.2.5; Ronquist *et al*., 2012). A total of 379 base pairs in three distinct regions of the alignment were not able to be unambiguously aligned, and were excluded from the analysis. Two runs of four chains each were run for 5 000 000 generations, saving every thousandth tree. Nodal posterior probabilities were assessed using the 50% consensus tree topology (Huelsenbeck *et al*., 2002), discarding the first 25% of trees as burnin. Aligned sequences and commands used in the phylogenetic analysis are provided in ***.nex (Supplementary Information).

Resulting forward and reverse sequences were aligned and manually edited to resolve any ambiguities in base calling. The resulting alignments were matched against the NCBI nucleotide database with BLASTn.

#### Statistics

A *χ*^2^ test was used to determine whether prevalence differences of parasites observed were significant (*P* values < 0.05) between sample sites in 2015. R packages used were dplyr, tidyr, ggplot2, car and NCStats.

## Results

### Histology

#### Cockles

**Fig. 2.**
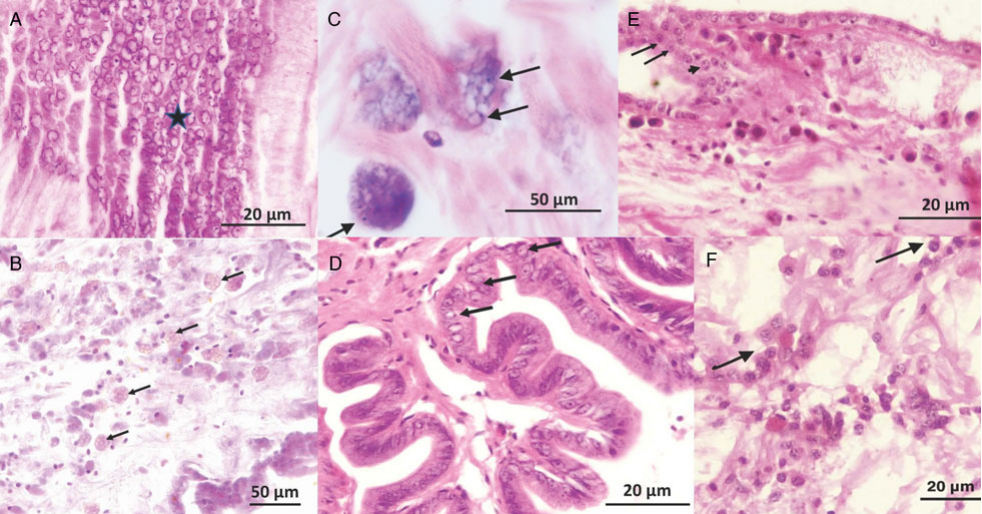
(A) Large number of haplosporidian spores in the connective tissues of *C. edule*, (B) & (C) multiple haplosporidia-like sporonts and developing spores respectively in the connective tissues of *C. edule*, (D) spores in the mantle tissue of *C. edule*, (E) uninucleate ‘fried egg’ (arrows) and binucleate (arrow head) cells in the epithelium of *C. edule* and (F) multiple plasmodia in the connective tissues of *C. edule*.

Haplosporidia-like single cell and plasmodia-like life stages identical to those described in Ramilo *et al*. (2017) and a spore-like stage (Fig. 2A–F) were observed in the connective tissues of Irish *C. edule* in a single individual at Flaxfort the nonculture site in June 2010 [0.11% (1/900)]. Subsequently in 2015, a subsample of PCR positive *C. edule* at Dungarvan (100% prevalence in the subsample of 10 PCR positive individuals) had positive detection of Haplosporidia-like cells in the corresponding histology section. Similarly in 2015 at Carlingford, Haplosporidia-like cells were observed in the tissues (mainly in the gill, gonad and digestive area, with some physical disruption to the tissues and infiltration of haemocytes) of a subsample of 20 PCR positive individuals (Table 1).

#### Mussels

In the *M. edulis*, haplosporidia-like single cell and plasmodia life stages similar to those described in the cockles were also observed in mussels deemed to be positive in the PCR (Clonea 1.7% (1/60) and Ringaskiddy 3.3% (2/60)). No spores were observed (Table 1).

Molecular genetic screening:

*Cockles*: A single cockle at Flaxfort Strand deemed to have haplosporidian-like cells visualized in the histology (0.11% prevalence, 1/900) also produced a PCR product (350 bp) (1.7% prevalence, 1/60) with the HAP-F1′/HAP-R3′ primers (Renault *et al*., 2000) in June 2010.

**Fig. 3.**
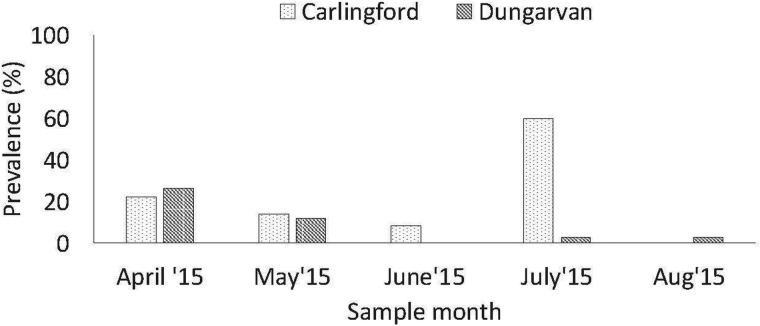
Prevalence of *M. mercenariae*-like parasite in *C. edule* at the two Irish *C. gigas* culture sites *from April to August 2015*.

Overall, 59 (8.3%, (59/704)) cockles produced a PCR product (350 bp) with the HAP-F1′/HAP-R3′ primers (Renault *et al*., 2000) from April to August 2015 at both oyster culture sites – the total prevalence was 4% (10/250) in Dungarvan and 10.8% (49/454) in the Carlingford cockles, which was statistically different (*χ*^2^ = 14.381, df = 1, *P* value < 0.001). The highest prevalence was detected in the Carlingford cockles in July and in the Dungarvan cockles in April (Fig. 3).

A single haplosporidian spp. *M. mercenariae-like* parasite in *C. edule* at Flaxfort Strand (2010) and at the *C. gigas* culture sites Dungarvan and Carlingford Lough (2015) was identified in the PCR products amplified in cockles from the three sites [Accession # KY522823.1 (Ramilo *et al*., 2017)] with 97% query coverage and 100% maximum identity and are referred to as ‘Isolates 221, 226 and 228’ in this study.

*Mussels*: No PCR products were produced using both primer pairs (Renault *et al*., 2000; Molloy *et al*., 2012) in the wild *Mytilus* spp. screened at the three culture sites at Dungarvan, Carlingford and Ballymacoda.

Overall, of the 11 nonculture sites screened two sites with 0.5%, (3/580) of mussels produced products (430 bp) using the ssu980/HAP-R1′ (Molloy *et al*., 2012). A total of 6.6% (2/60) of *M. edulis* from two Ringaskiddy samples [3.3% each, (1/30)] produced products (430 bp) in July 2017 and October 2017 respectively, while a single *M. edulis* (3.3%, 1/30) at Clonea in September 2017 produced a PCR product. All of these products were identified as Haplosporidia sp. SAL-2014 [Accession # KC852876.2 (Lynch *et al*., 2014)] with 98% query coverage and 100% maximum identity and are referred to as ‘Isolate 376’ in this study.

*Pacific oysters*:

No PCR products were produced using either primer pairs (Renault *et al*., 2000; Molloy *et al*., 2012) in the *C. gigas* screening or in the MSX-A′/MSX-B′ screening for *H. nelsoni* (Renault *et al*., 2000).

Direct sequencing:

The PCR product of the single mussel at Clonea was successfully sequenced however, the PCR products for both mussels at Ringaskiddy were also sent for sequencing but only the reverse sequences (which were a match) were amplified and not the corresponding forward sequence. As both sequences could not be aligned the result was not considered robust.

Due to the cost of sequencing all of the cockle PCR products amplified (*n* = 60), a subsample of cockle PCR products was sent for sequencing (representative of each sample site and years). Additionally, a similar morphology of each parasite was observed in the cockle and mussel histology for each respective parasite. More recent sequencing of PCR products (*n* = 40) from additional cockles at each location (study currently being carried out by the authors) has confirmed the findings of this study.

**Fig. 4.**
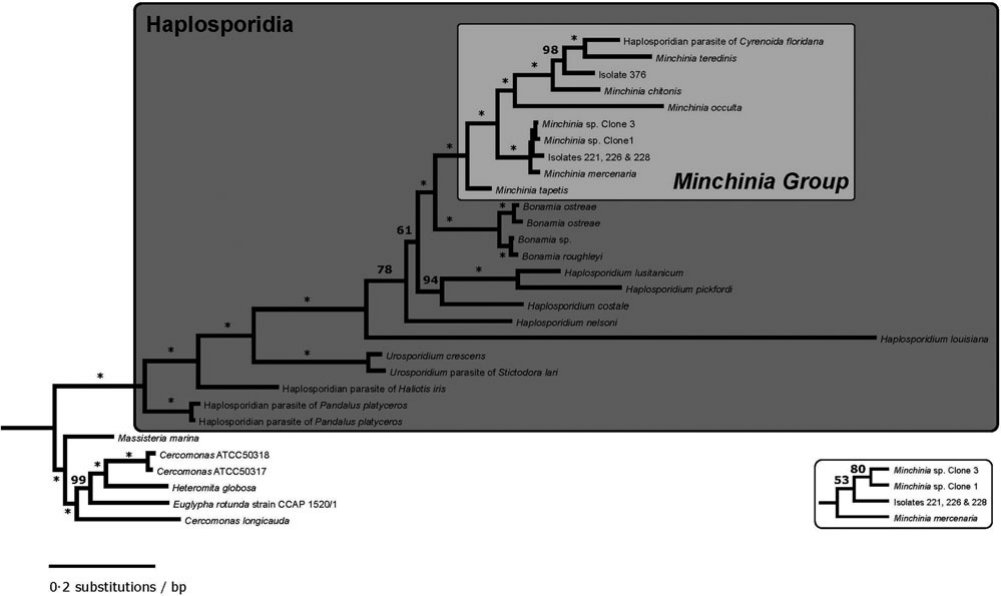
Bayesian phylogenetic tree of haplosporidian taxa based on partial 18S SSU rRNA sequences. Branches marked with an asterisk (*) have 100% posterior probability support for that node, otherwise, the nodal support is indicated by the number given. Inset to the bottom right repeats, for clarity, the *Minchinia* subclade with very small branch lengths, and gives the nodal posterior probabilities for this topology. A well-supported clade includes all of the 18S SSU rDNA sequences assigned to the genus *Minchinia*, the previously identified parasite of *C. floridana* (Reece *et al*., 2004), as well as the novel sequences for this study. As has been found in other analyses (Reece *et al*., 2004; Lynch *et al*., 2013; Ramilo *et al*., 2017), we find strong evidence for the paraphyly of the genus *Haplosporidium*.

Phylogenetic analyses:

The maximum likelihood phylogeny of haplosporidian taxa conclusively places Isolate 376, which is identical to the undescribed isolate SAL-2014 [Haplosporidia sp. Accession # KC852876.2 (Lynch *et al*., 2014)], and Isolates 221, 226 and 228, which are identical to *M. mercenariae-like* parasite [Accession # KY522823.1 (Ramilo *et al*., 2017)], in a clade with species of the genus *Minchinia* (Fig. 4). Also included in this clade is an undescribed haplosporidian parasite of the Florida marsh clam *C. floridana*. The internal topology of this clade is fairly well-resolved, with Isolate 376 and the *C. floridana* parasite being recovered with *M. chitonis* and *M. teredinis* with unanimous bootstrap support. The phylogeny also recovers unambiguous support for the monophyly of *Urosporidium* (Fig. 4). Several of the internal nodes of the phylogeny are poorly supported in the bootstrap (Fig. 4), although, these involve branching events among the *Bonamia* Group, the *Minchinia* Group and the paraphyletic genus *Haplosporidium.*

## Discussion

### Detection of Haplosporidia spp.

Two haplosporidian species were detected for the first time in *C. edule* and in *M. edulis* in a new geographic range. When detected, both Haplosporidia spp. were observed in individual samples of mussels and cockles that consisted of at least 30 individuals. The prevalence of the *M. mercenariae*-like parasite was significantly greater in *C. edule* at both aquaculture sites (late spring, summer and early autumn samples) compared to *C. edule* at the nonculture site (summer sample), which may indicate that the extended presence of this parasite has some association with anthropogenic inputs and activities in these areas. Oyster seed/spat consignments are routinely imported to both culture sites from France and the UK for on-growing in late spring. A low overall prevalence of the *M. mercenariae*-like parasite was detected in this study, which is similar to that observed in the Ramilo *et al*. (2017) study and for *M. mercenariae* infecting North American hard clams (Ford *et al*., 2009). Haplosporidia sp. SAL-2014 (Lynch *et al*., 2014), a novel species first detected in a Welsh *M. edulis* in 2012, was also detected in this study for the first time at a similar prevalence in wild Irish *M. edulis* at two nonculture sites. One of those sites is a busy ferry/shipping terminal in Cork Harbour and a ferry between Wales and Cork Harbour was in operation from the 1980s up until recently.

### Host partitioning

Interestingly the *M. mercenariae*-like parasite was not detected in the cohabiting *C. gigas* nor was it detected in the cohabiting *Mytilus* spp. Haplosporidia sp. SAL-2014 was exclusively detected in *M. edulis* even though *M. galloprovincialis* and *Mytilus* hybrids were present at the Irish sites where it was detected. This difference in emerging haplosporidian species detection, diversity and abundance in these three bivalve species strongly indicates that these parasite species are host specific and host partitioning is occurring. Additionally, the findings of this study would indicate that the haplosporidian species may be associated more with one environmental niche than another i.e. the sediment rather than with the water column for the *M. mercenariae*-like parasite, as the cockles were collected on the surface of the sediment and would normally be buried within the sediment similar to clams, while the oysters and mussels were at least 30 cm above the sediment on trestles or rocky outcrops respectively. Hartikainen *et al*. (2014) identified three new *Minchinia*-affiliated SSU-types in environmental samples at a single location at Weymouth, SW England, in 2011 and 2012. Two of the *Minchina* spp. were closely related to *M. mercenariae* while the third was identical to *M. tapetis* [both parasites were associated with Welsh cockle mortalities; Longshaw and Malham (2013)]. Hartikainen *et al*. (2014) observed that *Minchinia*-affiliated SSU-types were detected in water column samples, strongly indicating a planktonic life-cycle stage (predominantly in the 0.45–20-*μ*m size fraction) and were mostly in the April samples. Findings from this study and the Hartikainen *et al*. (2014) study would indicate that *Minchinia* spp. are niche selective or their detections are closely associated with their life stages i.e. in the water column in a planktonic intermediate host or near the sediment associate with primary bivalve host species.

### Phylogenetics

The phylogenetic analysis in this study recovers unambiguous support for the grouping of these two haplosporidian isolates into a single clade with members of the genus *Minchinia*. As such, the four isolates would support the detection of a new geographic range for both of these species within the genus *Minchinia*. This would represent a substantial increase to the known diversity of this genus, as only six species associated with host species are currently described. The low bootstrap support for the internal nodes in phylogeny involves the genus *Haplosporidium*, and the interrelationships between its species and the two main haplosporidian clades, the *Bonamia* Group and the *Minchinia* Group. *Haplosporidium* is paraphyletic (Reece *et al*., 2004), likely representing a plesiomorphic grade from which the remaining two clades derived. The short internal branch lengths and the low resolving power in the bootstrap potentially indicate a rapid diversification among haplosporidian taxa.

### Site effect and shore height influence

A higher prevalence of the *M. mercenariae*-like parasite was observed in *C. edule* at Carlingford compared to Dungarvan. Carlingford is a more sheltered site with a lot of shipping activity, while Dungarvan is an oceanic bay that experiences tidal flushing, greater water exchange and some shipping activity (Lynch *et al*., 2014; Bookelaar *et al*., 2018). Higher pathogen retention, prevalence and diversity occur at sheltered marine environments, as pathogens are less likely to be swept away on the tides (Lenihan, 1999; Lynch *et al*., 2016; Bookelaar *et al*., 2018). A higher prevalence of the *M. mercenariae*-like parasite was observed in *C. edule* at the high shore at Carlingford compared to cockles lower down the shore at the oyster trestles. *C. edule* higher up the shore may experience more stressful abiotic conditions such as air exposure, fluctuating temperatures, precipitation etc., which may make them more susceptible to infections (Wegeberg and Jensen, 2003) or it may be possible that *C. edule* at the high shore are more likely to be in contact with other host species (Lynch *et al*., 2014).

### Potential drivers of emerging parasites

It is not uncommon for parasites to be widespread in marine environments, especially along near shore coastlines (Raftos *et al*., 2014). Coastal marine environments are very vulnerable to climate change (Holt *et al*., 2010) and a changing marine environment can have a direct impact on the distribution, life cycle and physiological status of hosts, pathogens and vectors (Gallana *et al*., 2013). While a change in host, pathogen or vector does not necessarily translate into a change of the disease, it is the impact of climate change on the interactions between the disease components that impact disease risk (Gallana *et al*., 2013). In addition, natural coastal disturbance arising from storm surges and high energy systems, which are predicted to increase under future climate change conditions (IPCC, 2018), may also play their part in pathogen emergence. Storms are important episodic events that can resuspend and transport sediments and are known to cause large-scale advection (i.e. transfer of heat or matter), sediment resuspension and transport (Cacchione *et al*., 1987; Warner *et al*., 2008). An association between disease outbreaks in marine organisms and storm activity is recognized (Burge *et al*., 2014). In one study, a strong correlation with hurricane activity [and a strong storm (nor'easter)] and the amoebic pathogen *Paramoeba invadens*, causative agent of urchin disease in the green urchin *Strongylocentrotus droebachiensis* in the northwest Atlantic, was modelled and confirmed (Feehan *et al*., 2015). Other factors such as the movement of non-native species, both intentionally (i.e. for aquaculture) and unintentionally (i.e. as stowaways in ship ballast water or on hulls), brings the threat of ‘pathogen spillover’ into introduced areas and highly-susceptible host populations (Carnegie *et al*., 2016; Ek-Huchim *et al*., 2017). It is also recognized that coastal development may unbalance marine parasite-host systems (Coen and Bishop, 2015), as past emergent and resurgent diseases in wildlife appear to be associated with anthropogenic activities (Harvell *et al*., 1999; Daszak *et al*., 2000). Cultured bivalve breeding programmes are designed to mitigate the impacts of pathogens and may be unintentionally resulting in or expediating ‘host jumping’ from now less susceptible bivalve hosts to new cohabiting and highly susceptible naïve host species (Bookelaar *et al*., 2018). Such programmes may inadvertently be making selectively bred bivalve hosts more susceptible to novel pathogens. Additionally, due to the very poor biogeographical records available for protistan parasites it is possible that these parasites evolved in these hosts at those locations and were not introduced.

## Conclusions

The detection of both these Haplosporidia spp. in the *Minchinia* clade will contribute to an improved understanding of Haplosporidia diversity, prevalence, host, geographic distribution and a certain degree ecology. This study further supports that a *M. mercenariae*-like haplosporidan infects *C. edule* in Europe (Ramilo *et al*., 2017) and that it appears to be exclusive to *C. edule* while Haplosporidia sp. SAL-2014 (Lynch *et al*., 2014) appears to be exclusive to *M. edule*. As many other species, including protected bird species, rely on *C. edule* and *M. edulis* as a food source and the pivotal role both bivalve species play in marine coastal ecosystems, it would be beneficial to better understand the impact that these Haplosporidia spp. may or may not have on cockle and mussel populations currently and under future climate change conditions. Understanding some of the current drivers of parasite introduction, emergence and spread may facilitate a better prediction of future impacts and host or geographical range expansion of Haplosporidia under changing environmental scenarios. In particular with a parasite group such as the Haplosporidia, which have had such a devastating historical impact both commercially and ecologically.
